# The Dysregulation Profile Predicts Cannabis Use in the Offspring of Teenage Mothers

**DOI:** 10.1155/2013/659313

**Published:** 2012-11-11

**Authors:** Natacha M. De Genna, Cynthia Larkby, Marie D. Cornelius

**Affiliations:** Department of Psychiatry, University of Pittsburgh School of Medicine, 3811 O'Hara Street, Pittsburgh, PA 15213, USA

## Abstract

*Background*. Offspring of teenage mothers are at greater risk of early drug use. Research has identified a child behavior checklist (CBCL) profile for children with high levels of comorbid behavior problems, the dysregulation profile (DP), as another risk factor for drug use. *Method*. Teenage girls (12–18 years old; 71% African-American, 29% White) were recruited during pregnancy. Data were collected during pregnancy and when offspring were 6, 10, and 14 years old (*n* = 318). Mothers completed the CBCL when children were at ages 6 and 10, and children who scored 60 or higher on all 3 DP subscales (aggression, anxiety/depression, and attention problems) were categorized as dysregulated. At ages 10 and 14, the offspring (50% male, 50% female) reported on their cannabis use and completed the childhood depression inventory (CDI). *Results*. DP at age 6 and depressive symptoms at age 14 predicted recent cannabis use in the offspring. There was a significant interaction between race and pubertal timing such that White offspring who matured earlier were at greater risk of recent cannabis use. *Conclusions*. The results of this study suggest that it may be possible to identify a subset of children at risk of dual diagnosis as early as age 6.

## 1. Introduction

Researchers have identified a child behavior checklist (CBCL) profile for children with high levels of comorbid aggressive behavior, anxiety/depression, and attention problems, the Dysregulation Profile (DP). This profile, first identified by Biederman et al. [1], was initially considered junior or pediatric bipolar disorder and was labeled CBCL-PBD. More recently, however, the phenotype has been characterized as disruptive behavior disorders [2] and/or severe mood dysregulation and labeled DP [3]. DP appears to be highly genetic and relatively stable across childhood [4, 5]. The results of recent longitudinal studies suggest that children with DP are at greater risk of developing comorbid mood and substance use disorders by young adulthood [6–9]. 

There is a strong link between mood disorders and cannabis use during adolescence [10]. The effect appears to be unidirectional, with cannabis use preceding the onset of depressive symptoms [11]. In several longitudinal studies, adolescents who used cannabis were at greater risk of developing depression [11–14]. Fergusson and Horwood [15] reported that early use of cannabis (by age 16) predicted major depression by age 18 in a New Zealand birth cohort, even after controlling for adolescent mood disorder. Patton and colleagues found that daily adolescent cannabis use predicted the development of depression and anxiety in the next 7 years. In contrast, adolescent depression and anxiety did not predict cannabis use in this cohort. The results of these investigations suggest that early cannabis use may precede and even trigger depression in vulnerable individuals.

Data on depressive symptoms that precede cannabis use have provided evidence that cannabis use may increase risk for depression, even and perhaps especially in adolescents predisposed to depression [11]. Marmorstein et al. [16] demonstrated that girls from the Pittsburgh Girls Study in the “high depressive symptom” trajectory were more likely to suffer increases in depressive symptoms after initiating cannabis use. In the Raising Healthy Children Project, Fleming and colleagues [17] found that adolescents who increased substance use more rapidly than their peers were also more likely to experience increases in depressive symptoms. Based on this literature, cannabis use may be a precipitating factor for adolescent depression in vulnerable youth. Therefore, it is crucial to measure depressive symptoms in at-risk children prior to substance use to help elucidate the role of childhood and concurrent mood disorder on adolescent cannabis use. 

Dysregulated children enter adolescence at greater risk of developing a mood disorder. They may also be more predisposed to using cannabis during this period because of early association with deviant peers, problems with parents, and less attachment to academic achievement. The results of longitudinal studies on depression and cannabis use suggest that individuals are at increased risk of worsening their depressive symptoms if they initiate cannabis use during this key developmental period. However, it is not clear if childhood DP is associated with greater risk for cannabis use, above and beyond the risk for adolescent depression. More research is warranted on depression and early cannabis use in adolescents with and without a history of DP.

It is important to consider other possible covariates of early cannabis initiation, some of which may also be associated with childhood DP and adolescent mood disorder. For example, demographic factors such as race [18, 19] and socioeconomic status [20, 21] have been associated with different patterns of cannabis initiation. Early puberty has been linked to early drug use [22, 23] and depressive symptoms [24, 25]. Race, SES, and early maturation may influence cannabis initiation via association with substance-using peers, because peer substance use is a reliable predictor of early cannabis use [26–28]. Results from the Virginia Adult Twin Study of Psychiatric and Substance Use Disorders suggest that familial environmental factors have a powerful influence on substance use during early adolescence [29] so parental characteristics may also be an important component in cannabis initiation. In particular, there is good evidence that authoritative parenting can provide a buffer against adolescent substance use [28, 30, 31]. 

 The offspring of teenage mothers are at greater risk of behavior problems, including substance use, than the offspring of older mothers [32–35]. However, no studies to date have examined DP in the offspring of teenage mothers. D'Onofrio et al. [36] compared siblings from mothers of different ages in a nationally representative sample, concluding that children born to younger mothers were at greater risk of conduct problems such as cheating, telling lies, and destroying property. Harden et al. [37] similarly found that having a teenage mother predicted internalizing and externalizing behavior problems, using sibling and children-of-twins analyses. In both of these studies, the investigators were able to control for genetic and environmental confounds associated with having a young mother, to isolate the effect of younger maternal age from the effects of lower maternal education and being raised in a disadvantaged environment. However, it is unknown if there is a greater risk of DP in these offspring, and if childhood DP is a risk factor for their early cannabis use.

Higher rates of substance use have been documented in the offspring of teenage mothers compared to the children of older mothers, even after controlling for socioeconomic status (SES) and other factors associated with teenage motherhood. Fergusson and Woodward [34] found that 20% of the offspring of teenage mothers in the Christchurch Health and Development Study had a cannabis use disorder by age 18. After adjusting for SES, child rearing, child abuse, family functioning, and parental adjustment, this risk remained significantly higher for the offspring of younger mothers. Some longitudinal studies report gender differences in vulnerability. For example, Pogarsky and his colleagues [38] reported that the sons of teenage mothers in the Rochester Youth Development Study were at greater risk of drug use, but not the daughters. Many offspring of teenage mothers are resilient, however, and do not develop substance abuse problems [39, 40]. More research is needed to identify early determinants of risk for adolescent substance use in these high-risk offspring, including childhood DP. 

The data for this study are from the Teen Mother Study, a prospective study of teenage mothers and their children. The teenage mothers and their children were seen at delivery and at ages 6, 10, and 14. We hypothesized that DP measured at ages 6 and 10 would be associated with cannabis use by age 14, above and beyond the effects of depressive symptoms and other covariates identified in the literature on early substance use. These covariates included maternal characteristics such as the mother's age at the child's birth, family income, and parenting style. We also included child characteristics such as gender, depressive symptoms, early puberty, and early peer substance use. The goal of this study was to determine if childhood DP predicted cannabis use at age 14 in these high-risk children, even after controlling for depressive symptoms.

## 2. Materials and Methods

### 2.1. Procedure

Pregnant adolescents were recruited from the Magee-Womens Hospital prenatal clinic from 1990 to 1995. They were interviewed after a prenatal medical examination during their second trimester, and again at delivery with their newborn infants. Follow-up visits were conducted in our laboratory when the offspring were 6 (1995–2000), 10 (2000–2005), and 14 years old (2005–2010). The Institutional Review Boards of the Magee-Womens Hospital and the University of Pittsburgh approved each phase of this study. Staff was vigorously trained on all measures prior to each wave of assessment. Participants were informed about confidentiality and assured that their information was protected by a Certificate of Confidentiality issued by the National Institute on Drug Abuse (NIDA). 

 The first contact with the teenage mothers occurred before their fifth month prenatal visit. They were interviewed about their reproductive history and substance use in a private room at the prenatal clinic. At the 6-, 10-, and 14-year follow-up visits at our offices, the mothers provided information about their demographic and psychological status and recent substance use. Children were asked about peer substance use and reported on their own substance use at ages 10 and 14. Information from birth and offspring outcomes at ages 6 and 10 has been provided in previous reports (e.g., [33, 41–44]). The present study focuses on recent cannabis use in the 318 offspring seen at age 14.

All pregnant 12 to 18 year olds attending the prenatal clinic were eligible for the study. Of the 448 girls who were originally approached to participate, 3 girls refused. Of the remaining 445 pregnant teenagers, 15 moved out of the area prior to delivery, and 1 refused the delivery interview. Additional losses included six twin births, five spontaneous abortions, two still-born infants, and three live-born premature infants who died. Thus, 413 live-born singletons and their teenage mothers were assessed at delivery. A total of 318 women and their offspring were assessed at the 14-year follow-up phase, 77% of the birth cohort. Twenty-three mothers refused to participate, 54 were lost to follow-up, 9 had moved out of the state, 2 children were adopted, and 7 children had died. 

Out of the 318 completers, data for all measures in the current study were available for 254 mother-child dyads. Children who were seen at all 4-time points were included in this analysis. Missing data were examined using Little's test [45] and determined to be missing completely at random (MCAR). This test compares participants with and without missing data using *t*-tests to determine if they are significantly different with an alpha = .05. Prenatal substance use and SES did not differ significantly between the mothers and offspring who completed the age 14 followup and those from the original birth cohort who were not assessed during this wave. 

### 2.2. Participants

The mothers, on average, were 16.3 years old (range = 12–18 years old) at study recruitment: 75% were at least 16 years old, and 25% were 12–15 years old. Seventy-one percent are African-American, 29% are White, and 99% of them were unmarried at delivery [41]. At the 14-year followup, the average monthly family income was $2,253 (range = $0–$9,990), and mean maternal education was 12.8 years (range = 7–18 years). Most of the mothers (73%) had completed high school or received a General Equivalency Diploma (GED), 5% completed college. Most of the mothers (72%) were not currently married. 

The offspring, on average, were 14.5 years old (SD = 0.6: range = 13–16 years old) at the last wave of testing. This sample included 158 daughters and 160 sons. Most of the children (84%) lived with their biological mothers at the age 14-year followup; the remaining 16% of the children lived with a custodian. The current custodian was interviewed for the study, so in these cases maternal responses reflect the responses of the current maternal figure for the child. A variable indicating that the child was not in his or her biological mother's custody at the age 14 follow-up visit was not significantly associated with behavioral dysregulation at ages 6 or 10 (Φ = −.04 and −.03, resp.), adolescent depression (Φ = .08), or recent adolescent cannabis use (Φ = .07) and was dropped from further analysis. 

### 2.3. Measures

#### 2.3.1. Childhood Behavioral Dysregulation Profile

Caregivers completed the child behavior checklist (CBCL: [46]) during the age 6 and age 10 assessments. *T*-scores equal to or higher than 60 on the attention problems, anxious/depressed, and the aggressive behavior subscales were used to calculate the behavioral dysregulation profiles at ages 6 and 10, resulting in a dichotomous measure indicating that the child met criteria for DP. The results of longitudinal studies on DP suggest that it remains relatively stable across childhood [4, 5]. In the current study, DP at age 6 was significantly correlated with DP at age 10. Four percent of the offspring met criteria for DP at the age 6 assessment, and 6% of the sample met DP criteria at age 10. Meyer and her colleagues [9] examined long-term sequelae of these subthreshold levels of comorbidity on the 3 subscales in children from a high-risk community sample. The authors report that children with the behavioral dysregulation profile using this cutoff were significantly more likely to suffer from comorbid psychopathology by young adulthood.

#### 2.3.2. Adolescent Depression

 Adolescent depression was measured using the child depression inventory (CDI: [47]), a self-report measure with 27 items. Total *T*-scores for the sample from 35–94 (*M* = 46.21, SD = 9.33) at age 10 and 34–93 (*M* = 44.44, SD = 8.53) at age 14. Based on the guidelines provided in the CDI manual, 8% of the offspring at age 10 and 10% of the offspring at age 14 had depressive symptom *T*-scores that could be considered “above average.” The *T-*scores at ages 10 and 14 were only modestly correlated (*r* = .12, *P* < .05), and therefore, both variables were included in the multivariate analysis.

#### 2.3.3. Recent Cannabis Use

Adolescents were asked “In the past year, on the days when you used cannabis, about how many joints did you usually smoke?” This was used to calculate average daily joints. The outcome measure in this study is any cannabis use in the past year (0: average daily joints = 0, 1: average daily joints > 0). Fifty-one offspring (16% of the sample) used cannabis in the past year. There were no racial or gender differences in recent cannabis use among the adolescents.

#### 2.3.4. Early Peer Drug Use

Peer cigarette, alcohol, and cannabis use were measured at age 10. The offspring were asked what proportion of their friends used each substance: none, some, most, or all. These variables were then dichotomized (0 = none, 1 = some/most/all). Thirty-six percent of the offspring reported that their peers were smoking cigarettes by age 10. A similar percentage of the offspring (37%) reported that their peers used alcohol by age 10, but this variable was not significantly associated with behavioral dysregulation at age 6 (Φ = −.05) or age 10 (Φ = −.05) or with cannabis use at age 14 (Φ = .09). Only 4% of the offspring reported that their peers had ever used cannabis by age 10. Peer cannabis use at age 10 was not significantly associated with behavioral dysregulation at age 6 (Φ = −.05) or age 10 (Φ = .08) or with recent cannabis use at age 14 (Φ = −.05). Hence, peer alcohol and cannabis use by age 10 were not included in the multivariate analysis.

#### 2.3.5. Pubertal Timing

This was a self-report measure. Adolescents were asked to compare their pubertal development to same-sex and same-age peers by answering this question from the Petersen development scale: “Do you think your development is any earlier or later than most other boys/girls your age?” The possible responses were as follows: (1) much earlier than others your age, (2) a little earlier than others your age, (3) about the same as others your age, (4) a little later than others your age, (5) and much later than others your age [48]. This ordinal measure was trichotomized into “early” for children responding 1-2, “on time” for children who responded 3, and “late” for children responding 4-5 to the pubertal timing question. Half the offspring (51%) that answered this question (*n* = 310) rated themselves as “about the same as others their age,” with 22% rating themselves as “a little earlier,” and 7% rating themselves as “much earlier” than others their age. Fewer adolescents rated their pubertal development as “a little later” (13%) and “much later” (5%) than their peers. There were no significant gender differences in pubertal timing. For example, 29% of girls and 30% of boys reported that they were “a little” or “much earlier” than their same-age peers. 

#### 2.3.6. Authoritative Parenting

Authoritative parenting was assessed with the “My Parents” questionnaire, which has 27 items measuring 3 dimensions of parenting: parental acceptance and involvement, autonomy-granting, and strictness-supervision [49]. The parental involvement scale (alpha = .72) included 15 questions asking adolescents how strongly they agreed or disagreed with items such as “I can count on my parent(s) to help me out, if I have some kind of problem” and “My parent(s) know who my friends are.” The autonomy-granting scale (alpha = .82) includes 12 items such as “my parent(s) let me make my own plans for things I want to do” and “My parent(s) tell me that their ideas are correct, and that I should not question them.” The strictness-supervision scale (alpha = .76) includes 9 items such as “In a typical week, what is the latest that you can stay out on week nights (Sunday–Thursday)?” and “How much do your parents really know about where you go at night?” Scores from these 3 parenting scales were summed to create composite scores. An ordinal authoritative parenting score (range = 0–3, *M* = 1.4, SD = 0.9) was then generated using the method described by Steinberg and colleagues [49]. Scores above the sample median on the involvement, autonomy, and strictness-supervision scales were coded “3” for authoritative parenting. Scores below the sample median on all 3 dimensions of parenting were assigned an authoritative parenting score of “0.” Scores above the sample median on one or two dimensions received authoritative parenting scores of “1” or “2.”

#### 2.3.7. Demographic Variables

Several demographic variables were included in the multivariate analysis on recent cannabis use, including race of mother, age of mother during the index pregnancy, sex of adolescent, current age of adolescent, and family income. Mothers reported their age and race at the time of entry into the Teen Mother Study. Offspring sex was recorded at delivery. The child's birth month and year was used to calculate age at the 14-year assessment. Child age was included in the multivariate analyses to control for the age range among offspring and the effect of chronological age on risk for recent cannabis use. Monthly family income during childhood was measured by maternal report when the offspring were 6 and 10 years old. Average monthly family income was $1356 at age 6 (SD = 1,138) and $1,789 at age 10 (SD = 1,454). Family income from the age 6 and age 10 assessments was averaged to create childhood family income scores (*M* = 1,570, SD = 1,154). 

### 2.4. Statistical Analyses

 Descriptive statistics were conducted on the variables of interest in the study. A hierarchical logistic regression analysis was then used to determine if childhood DP predicted recent cannabis use in 14-year-old offspring, after controlling for covariates of early cannabis initiation that have been previously identified in the literature. There was sufficient power to include 13 predictors in the final model with this sample size [50]. Predictors were entered in chronological order, beginning with demographic characteristics. Next, DP status at ages 6 and 10 was entered into the equation. The following steps were peer substance use at age 10 and depression scores at age 10. Age 14 covariates in the next step included depression scores, authoritative parenting, and pubertal timing. In the last step, we tested for interaction effects between race and each predictor, because of evidence that there may be different risk factors for health-risk behavior in White and African-American children (e.g., [51–53]).

## 3. Results

### 3.1. Bivariate Analyses

Descriptive statistics for the predictors of recent cannabis use are presented in Table 1, including the mean, standard deviation, and range of continuous variables. Percentages are provided for dichotomous variables. Intercorrelations among DP at ages 6 and 10, the other predictors, and cannabis use at age 14 are presented in Table 2. The behavioral dysregulation profiles at ages 6 and 10 were not highly correlated (Φ = .24), and their coefficients in the regression analysis were not correlated highly enough to indicate multicollinearity (Φ = −.47). Therefore, both variables were used in the multivariate analysis predicting adolescent recent cannabis use. Younger maternal age, older adolescent age, lower childhood family income, less authoritative parenting, and concurrent adolescent depressive symptoms were significantly correlated with cannabis use at age 14. Neither race nor sex was associated with cannabis use in this cohort.

### 3.2. Multivariate Analysis

**Figure 1:**
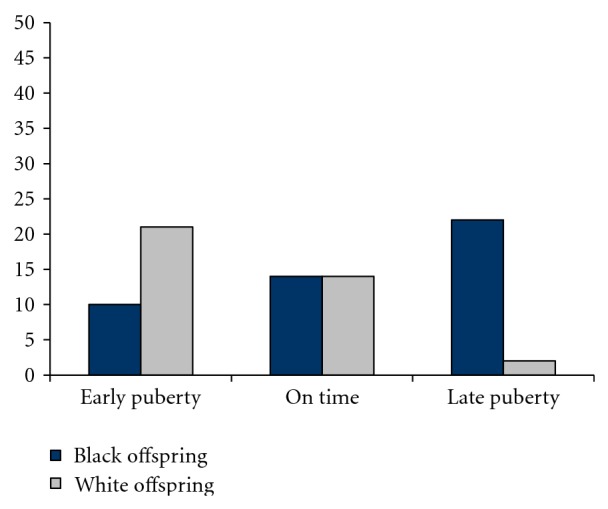
Percentage of teen offspring who reported recent cannabis use as a function of race and pubertal timing.

 The results of the hierarchical logistic regression analysis predicting recent cannabis use in the 14-year-old offspring are presented in Table 3, which illustrates the final step of the equation (*χ* ^2^ = 49.5, *P* < .001, Cox & Snell *r* ^2^ = .19). DP at age 6, older adolescent age, and higher depression scores were significant predictors of recent cannabis use. There was also a significant interaction of race and pubertal timing (Figure 1). There was a linear effect of pubertal timing on recent cannabis use for the White offspring, such that those who matured earlier were more likely to have recently used cannabis. By contrast, the African-American offspring who matured later appeared to be at greater risk of recent cannabis use than African-American offspring who matured earlier than their peers. 

## 4. Discussion

This was the first study to examine DP in offspring of teenage mothers and to associate childhood DP with early cannabis use in these vulnerable adolescents. DP at age 6 was a significant predictor of cannabis use at age 14, suggesting that it may be possible to identify children at risk of substance abuse at an early age within this vulnerable group. DP was correlated across middle childhood in this cohort, consistent with Althoff et al. [4]. However, DP at age 10 did not predict cannabis use by age 14, contrary to our hypotheses. It is possible that behavioral dysregulation at age 6 may be especially detrimental for behavioral risk, at age of entry into the school system. This is a developmental period when children become independent from their families and spend their entire day at school, learning to concentrate on new cognitive tasks and avoid disrupting the classroom. They must also learn to form and nurture peer relationships that are less managed by their families while at school. Children who fail to meet these challenges at a young age may be especially vulnerable to early cannabis use compared to children who only become extremely dysregulated by age 10. 

 Age 6, DP was also associated with having more peers who used cigarettes by age 10, suggesting that one potential pathway from early dysregulation to early cannabis use is association with deviant peers. However, there was a separate independent effect of age 6 DP on cannabis use at age 14, after controlling for the effects of age 10 peer cigarette use and age 14 depressive symptoms, indicating an additive effect of childhood DP on risk for adolescent cannabis use. This is an important finding and provides converging evidence that DP is a risk factor for substance use in high-risk community samples, and not just clinical samples of children and adolescents. Future work will examine the effect of DP on early tobacco and alcohol use, to determine if childhood DP predicts other substance use in this vulnerable group of adolescents. There is evidence that early cannabis use predisposes individuals to developing a substance use disorder by young adulthood in general, and not just a cannabis use disorder specifically [54]. Additionally, we will examine the effects of childhood DP on these substances at age 16, testing to determine if DP continues to predict cannabis and other substance use a more normative age.

This study contributes to the literature on long-term behavioral risk in the adolescent children of teenage mothers. There are several reasons to expect a higher prevalence of DP in the offspring of teenage mothers than in the general population, including evidence of a strong genetic component [5]. Aside from genetic loading, the children of teenage mothers may experience different rearing environments than children of older mothers, which may also contribute to behavioral dysregulation [55]. For example, they are more likely to be reared in poverty [56, 57] and in less optimal home environments [58, 59]. Although 48 children were no longer in maternal custody at age 14, custody was not associated with DP at ages 6 and 10. Reports of children's behavior problems may also be influenced by maternal psychopathology [60], and teenage mothers may experience more mental health problems than women who delay childbearing [61, 62]. The present findings suggest that a subsample of children of teenage mothers at extremely high risk of early cannabis use may be identified by elementary school.

It is difficult to compare DP across studies because different criteria are used to characterize DP, and samples vary considerably with respect to mental health and age range. Hudziak et al. [63] report less than 1% in a Dutch twin sample at ages 7 and 10. Volk and Todd [2] found DP in 2.5% of children from a twin study of ADHD. In both of these studies, the investigators used *T*-scores greater than 70 as the cutoff for DP. We used the more liberal cutoff score of 60 proposed by Meyer and colleagues [9] for nonclinical samples and found that 4% and 6% of our cohort met criteria for DP at ages 6 and 10, respectively. Although Meyer et al. reported that 16% of their sample was classified with DP using *T*-scores ≥ 60; two-thirds of the mothers of these children had a mood disorder. Only 6% of the children from the Meyer et al. sample whose mothers did not have a mood disorder (*n* = 1) met criteria for DP. Based on these comparisons, it is impossible to conclude that the offspring of teenage mothers are at greater risk of DP. 

Although this study was unique and contributes to our knowledge of risk for cannabis use in offspring of teenage mothers, there were some limitations. DP was established using caregiver report, as in previous studies of DP. However, Althoff and colleagues [64] found that agreement across informants such as parents, youths, and teachers is only mild to fair. Thus, it is possible that their caregivers may have misclassified some youth. Another limitation was the relatively small sample size, with only 12 children meeting criteria for DP at age 6, and 17 children meeting criteria at age 10. Therefore, similar to the Meyer et al. [9] study, we needed to limit the inclusion of potential predictors in our analyses. Another limitation shared with Meyer et al. [9] was our use of the less stringent “community sample” DP criteria. Thus, our results may not generalize to clinical samples, where the more stringent cutoffs are applied. Finally, we did not include a biological measure for cannabis use. However, use at age 14 is likely to be sporadic and the half-life for THC is only 12–24 hours [65]. Biological measures may miss a significant amount of use. To increase the accuracy of the reported data, we constructed detailed questions, thoroughly tested our measures, carefully selected interviewers, and extensively trained our staff in interview techniques. 

## 5. Conclusions

 This is the first study to examine DP in the offspring of teenage mothers and to consider childhood DP as a predictor of cannabis use during adolescence. It is not yet clear if DP is more prevalent in the offspring of teenage mothers. Our finding that this phenotype is associated with cannabis use by age 14 in this sample adds to a converging line of evidence that DP is a risk factor for substance abuse [6, 7, 9]. DP at age 6 remained a significant predictor of recent cannabis use at age 14 after adjusting for depressive symptoms at age 10 and concurrent depressive symptoms, suggesting that DP has predictive validity for early cannabis use above and beyond its association with depression. One of the implications of this study is that early identification of children with DP may help target interventions to prevent early cannabis use in this vulnerable population, thereby obstructing one pathway to dual diagnosis by adulthood. These children should be monitored for a long-term for risky behavior and association with deviant peers, even if they display less depressive and other behavioral symptoms as they enter adolescent. Another clinical implication is that early puberty may be an important risk factor for early use in White but not African-American children.

## Figures and Tables

**Table 1:** Descriptive statistics: predictors of recent adolescent cannabis use.

	Percentage	Mean (standard deviation)	Range
(1) Race of mother (White)	29%		
(2) Age of mother at time 1 (years)		16.3 (1.2)	12–18
(3) Sex of adolescent (male)	50%		
(4) Age of adolescent		14.5 (0.6)	13–16
(5) Childhood family income^1^		1569 (1154)	158–8,000
(6) Dysregulation profile (DP) at age 6	4%		
(7) Dysregulation profile (DP) at age 10	6%		
(8) Peer cigarette use at age 10	36%		
(9) Depression scores at age 10		46.2 (9.3)	35–94
(10) Depression scores at age 14		44.4 (8.5)	34–93
(11) Authoritative parenting at age 14		1.4 (0.9)	0–3
(12) Pubertal timing		2.9 (0.9)	1–5

^*^ *P* < .05, ∗∗*P* < .01.

^1^Mean monthly family income from age 6 and age 10 assessments.

**Table 2:** Intercorrelations among predictors and recent adolescent cannabis use.

	1	2	3	4	5	6	7	8	9	10	11	12	13
(1) Race of mother (White)	—	.14∗	−.05	.13∗	.21∗∗	−.05	−.03	−.06	−.02	.07	.01	−.04	.01
(2) Age of mother at time 1	—	—	−.02	.04	.22∗∗	−.20∗∗	−.08	.01	.08	−.04	.04	.04	−.11∗
(3) Sex of adolescent (male)	—	—	—	.04	.05	.00	.01	.09	−.10	−.10	−.04	.05	−.01
(4) Age of adolescent	—	—	—	—	.09	−.04	−.08	.08	−.05	.09	−.03	.02	.19∗∗
(5) Childhood family income^1^	—	—	—	—	—	−.10	−.01	−.11	−.03	−.09	.09	.04	−.15∗
(6) Dysregulation profile (DP) at age 6	—	—	—	—	—	—	.24∗∗	.13∗	−.07	.01	−.06	−.04	.05
(7) Dysregulation profile (DP) at age 10	—	—	—	—	—	—	—	.06	−.06	.03	−.06	.00	−.06
(8) Peer cigarette use at age 10	—	—	—	—	—	—	—	—	.00	.05	−.04	.00	.03
(9) Depression scores at age 10	—	—	—	—	—	—	—	—	—	.12∗	−.07	.13∗	−.02
(10) Depression scores at age 14	—	—	—	—	—	—	—	—	—	—	−.36∗∗	−.07	.27∗∗
(11) Authoritative parenting at age 14	—	—	—	—	—	—	—	—	—	—	—	−.09	−.20∗∗
(12) Pubertal timing	—	—	—	—	—	—	—	—	—	—	—	—	.04
(13) Recent adolescent cannabis use	—	—	—	—	—	—	—	—	—	—	—	—	—

^*^ *P* < .05, ∗∗*P* < .01.

^1^Mean family income from age 6 and age 10 assessments.

**Table 3:** Final step of logistic regression model predicting recent cannabis use (*n* = 263).

Predictors	Beta	S.E.	Wald	Exp (B)	Confidence intervals
Race of mother (White)	2.64	0.87	9.31∗∗	14.0	2.57–76.2
Age of mother at time 1	−0.17	0.20	0.72	0.85	0.57–1.25
Sex of adolescent (male)	0.41	0.48	0.76	1.51	0.60–3.83
Age of adolescent	1.23	0.37	11.31∗∗	3.42	1.67–7.01
Childhood family income^1^	−0.03	0.02	1.91	0.97	0.93–1.01
Dysregulation profile (DP) at age 6	2.11	0.94	4.99∗	8.23	1.30–52.3
Dysregulation profile (DP) at age 10	−2.08	1.45	2.06	0.13	0.01–2.15
Peer cigarette use at age 10	−1.00	0.54	3.47	0.37	0.13–1.05
Depression scores at age 10	0.00	0.03	0.00	1.00	0.95–1.06
Depression scores at age 14	0.07	0.03	5.12∗	1.07	1.01–1.13
Authoritative parenting at age 14	−0.56	0.30	3.52	0.57	0.32–1.03
Pubertal timing	0.93	0.39	5.74∗	2.54	1.18–5.44
Pubertal Timing × Race	−2.82	0.91	9.70∗∗	0.06	0.01–0.35

^*^ *P* < .05, ∗∗*P* < .01.

^1^Mean family income from age 6 and age 10 assessments.
